# Use of Physcion to Improve Atopic Dermatitis-Like Skin Lesions through Blocking of Thymic Stromal Lymphopoietin

**DOI:** 10.3390/molecules24081484

**Published:** 2019-04-15

**Authors:** Phil-Dong Moon, Na-Ra Han, Jin Soo Lee, Sungwei Hong, Min-Sun Yoo, Hyeong-Jin Kim, Ji-Hyeon Kim, Soonsik Kang, Hyun-Woo Jee, Hyung-Min Kim, Hyun-Ja Jeong

**Affiliations:** 1 Department of Pharmacology, College of Korean Medicine, Kyung Hee University, Seoul 02447, Korea; pdmoon@khu.ac.kr (P.-D.M.); nrhan@khu.ac.kr (N.-R.H.); mcjin21@naver.com (J.S.L.); 2 Center for Converging Humanities, Kyung Hee University, Seoul 02447, Korea; 3 Department of Science in Korean Medicine, Graduate School, Kyung Hee University, Seoul 02447, Korea; lov4all@hanmail.net (S.H.); ymshappy@nate.com (M.-S.Y.); khjhj0999@hanmail.net (H.-J.K.); sjsdlsrk@naver.com (J.-H.K.); sensedatum@naver.com (S.K.); aidenjee@naver.com (H.-W.J.); 4 Department of Food Science & Technology and Research Institute for Basic Science, Hoseo University, Chungnam 31499, Korea

**Keywords:** physcion, thymic stromal lymphopoietin, atopic dermatitis, mast cells, caspase-1

## Abstract

Physcion is well known for the treatment of carcinoma. However, the therapeutic effect of physcion on atopic dermatitis (AD) through the inhibition of thymic stromal lymphopoietin (TSLP) level remains largely unknown. In this study, we investigated the anti-AD effect of physcion using HMC-1 cells, splenocytes, and a murine model. Treatment with physcion decreased production and mRNA expression levels of TSLP, IL-6, TNF-α, and IL-1β in activated HMC-1 cells. Physcion reduced the expression levels of RIP2/caspase-1 and phospho (p)ERK/pJNK/pp38 in activated HMC-1 cells. Physcion suppressed the expression levels of pIKKβ/NF-κB/pIkBα in activated HMC-1 cells. Moreover, physcion attenuated the production levels of TSLP, IL-4, IL-6, TNF-α, and IFN-γ from activated splenocytes. Oral administration of physcion improved the severity of 2,4-dinitrochlorobenzene-induced AD-like lesional skin through reducing infiltration of inflammatory cells and mast cells, and the protein and mRNA levels of TSLP, IL-4, and IL-6 in the lesional skin tissues. Physcion attenuated histamine, IgE, TSLP, IL-4, IL-6, and TNF-α levels in serum. In addition, physcion inhibited caspase-1 activation in the lesional skin tissues. These findings indicate that physcion could ameliorate AD-like skin lesions by inhibiting TSLP levels via caspase-1/MAPKs/NF-kB signalings, which would provide experimental evidence of the therapeutic potential of physcion for AD.

## 1. Introduction

Atopic dermatitis (AD) is one of the most common relapsing inflammatory skin diseases in the world [1]. AD has an estimated prevalence of 15–20% in children and 2.6–8% in adults worldwide [2,3]. AD worsens quality of life and results in considerable burdens such as insufficient sleep, school and/or work absenteeism, psychological stress, and high medical costs [4].

Proinflammatory cytokine thymic stromal lymphopoietin (TSLP) is perceived as a pivotal factor in the pathogenesis of atopic diseases such as AD and allergic rhinitis. Many studies have reported that application of 2,4-dinitrofluorobenzene (DNFB) results in AD-like skin lesions in mice and that TSLP expression is remarkably elevated in AD murine models [5,6,7]. Mast cells have an important role in atopic disorders [8]. Infiltration and activation of mast cells were upregulated in various AD animal models, indicating a contribution of mast cells in AD [9,10,11].

Although most caspases are usually involved in programed cell death, caspase-1 plays a role in inflammatory reactions [12,13]. Deletion of caspase-1 improved intestinal inflammation induced by dextran sulfate sodium [14]. The caspase-1/nuclear factor (NF)-κB signal cascade controlled TSLP expression in HMC-1 cells [15]. Furthermore, activation of NF-κB was downregulated by treatment with caspase-1 inhibitor, suggesting that NF-κB is a downstream molecule of caspase-1 [15].

**Figure 1:**
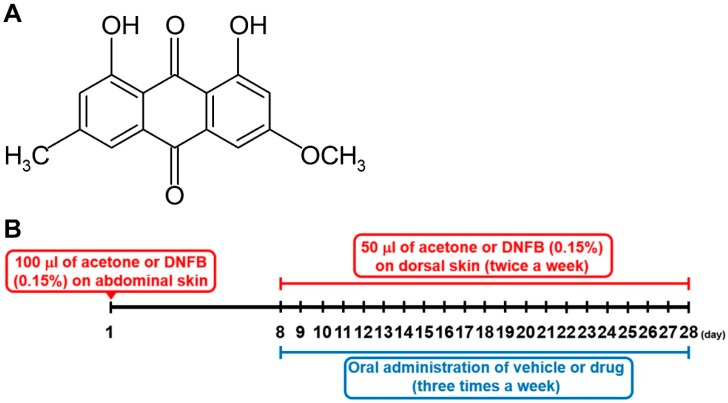
(**A**) Chemical structure of physcion. (**B**) Schematic protocol of a 2,4-dinitrofluorobenzene (DNFB)-induced atopic dermatitis (AD)-like murine model.

Physcion (1,8-dihydroxy-3-methoxy-6-methylanthraquinone, Figure 1A), a natural anthraquinone derivative, is present in vegetables such as cabbage lettuce and beans [16]. Physcion has various pharmacological properties, such as anti-inflammatory, anti-microbial, and anti-tumor [17]. However, most research on physcion are about tumors and apoptosis [18,19,20]. The inhibitory effects of physcion on TSLP levels from HMC-1 cells as well as on AD-like skin lesions have not been determined. Thus, in this study, we investigated whether physcion could downregulate the TSLP level in human mast cell line, HMC-1 cells and mice.

## 2. Results

### 2.1. Physcion Attenuates TSLP Level in PMA Plus Calcium Ionophore (PMACI)-Stimulated HMC-1 Cells

**Figure 2:**
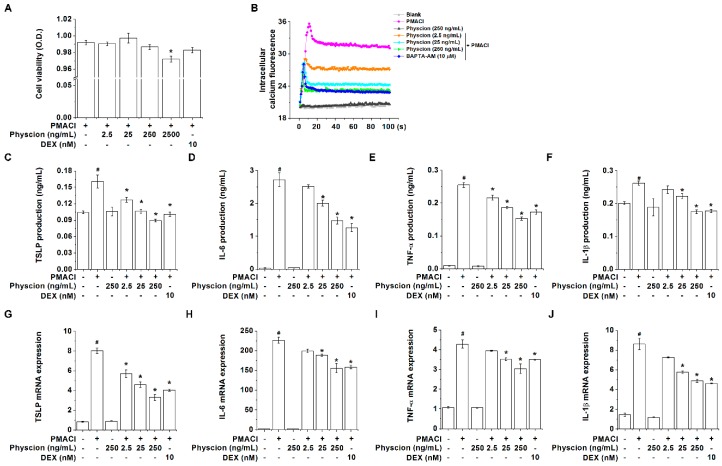
Physcion attenuates thymic stromal lymphopoietin (TSLP) levels in PMA Plus Calcium Ionophore (PMACI)-stimulated HMC-1 cells. (**A**) HMC-1 cells were treated with Physcion or Dexamethasone (DEX) for 1 h and then stimulated with PMACI for 7 h. Cell viabilities were analyzed with a MTT assay. (**B**) Physcion or BAPTA-AM was treated in Fura-2/AM-pretreated HMC-1 cells for 20 min. The HMC-1 cells were then stimulated with PMACI. Blank, non-stimulated cells; PMACI, PMACI-stimulated cells. HMC-1 cells were treated with Physcion or DEX for 1 h and then stimulated with PMACI for 7 h for ELISA or 5 h for polymerase chain reaction (PCR). The production levels of (**C**) TSLP, (**D**) IL-6, (**E**) TNF-α, and (**F**) IL-1β were detected by ELISA. The mRNA expression levels of (**G**) TSLP, (**H**) IL-6, (**I**) TNF-α, and (**J**) IL-1β were detected by Quantitative real-time PCR. Glyceraldehyde-3-phosphate dehydrogenase (GAPDH) mRNA expression levels were analyzed as a housekeeping gene for normalization. Data is expressed as fold induction relative to a vehicle group. #*p* < 0.05 vs. a non-stimulated group. * *p* < 0.05 vs. a PMACI-stimulated group.

First, we determined the concentration of physcion that did not affect the viability of HMC-1 cells. Figure 2A shows that 2500 ng/mL of physcion had significant cytotoxicity to PMACI-stimulated HMC-1 cells (*p* < 0.05). Thus, we determined physcion concentrations at 2.5, 25, and 250 ng/mL in this study. In our previous study, we reported that TSLP secreted from mast cells played a pivotal role in the pathogenesis of AD [10]. The TSLP secreted from mast cells is regulated by intracellular calcium [21]. Thus, we clarified whether physcion could regulate TSLP levels by reducing the intracellular calcium in mast cells. As shown in Figure 2B, the stimulation with PMACI markedly increased intracellular calcium levels. However, the treatment with physcion suppressed the intracellular calcium levels (Figure 2B). The beneficial effect on the calcium level of physcion (250 ng/mL)-treated group was similar to that of 2-bis(2-aminophenoxy)ethane-N,N,N0,N0-tetraacetic acid acetoxymethyl ester (BAPTA-AM, calcium chelator)-treated group. Next, we examined the regulatory effect of physcion on TSLP levels secreted from the PMACI-stimulated HMC-1 cells. The treatment with physcion (2.5, 25, and 250 ng/mL) significantly induced dose-dependent reductions in the production and mRNA expression levels of TSLP in the PMACI-stimulated HMC-1 cells (Figure 2C,G, *p* < 0.05). In addition, physcion (25 and 250 ng/mL) significantly suppressed the production and mRNA expression levels of IL-6, TNF-α, and IL-1β (Figure 2D–F,H–J, *p* < 0.05). Physcion alone did not affect these levels in non-stimulated HMC-1 cells (Figure 2). Dexamethasone (DEX) also had a regulatory effect on the TSLP, IL-6, TNF-α, and IL-1β levels in activated HMC-1 cells (Figure 2C–J, *p* < 0.05).

### 2.2. Physcion Downregulates RIP2 and Caspase-1 Expressions in PMACI-Stimulated HMC-1 Cells

**Figure 3:**
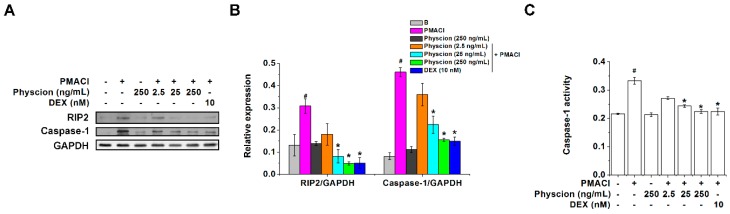
Physcion down-regulates RIP2 and caspase-1 expressions in PMACI-stimulated HMC-1 cells. (**A**) HMC-1 cells (5 × 10^6^) were treated with physcion or DEX for 1 h and then stimulated with PMACI for 30 min. Representative immunoblots are shown. (**B**) Each relative intensity of protein levels was quantified by densitometry and shown as RIP2/GAPDH and caspase-1/GAPDH. (**C**) Caspase-1 activity was determined with a caspase-1 assay kit. #*p* < 0.05 vs. a non-stimulated group. *p < 0.05 vs. a PMACI-stimulated group.

In our previous reports, RIP2 and caspase-1 regulated TSLP levels in HMC-1 cells [15,21]. Thus, we examined the expression levels of RIP2 and caspase-1 in the PMACI-stimulated HMC-1 cells to clarify the underlying mechanisms of the regulatory effect of physcion on TSLP levels. As shown in Figure 3A,B, PMACI stimulation significantly increased the expression levels of RIP2 and caspase-1 (*p* < 0.05) however, the treatment with physcion (25 and 250 ng/mL) significantly decreased the expression levels of RIP2 and caspase-1 (*p* < 0.05). In addition, physcion (25 and 250 ng/mL) significantly suppressed caspase-1 activities in the PMACI-stimulated HMC-1 cells (Figure 3C, *p* < 0.05). The regulatory effects of physcion (250 ng/mL) on expression levels of RIP2 and caspase-1 were similar to those of the DEX-treated group (Figure 3).

### 2.3. Physcion Attenuates Phospho (p)ERK/pJNK/pp38 And pIKKβ/NF-κB/pIkBα Expressions in PMACI-Stimulated HMC-1 Cells

**Figure 4:**
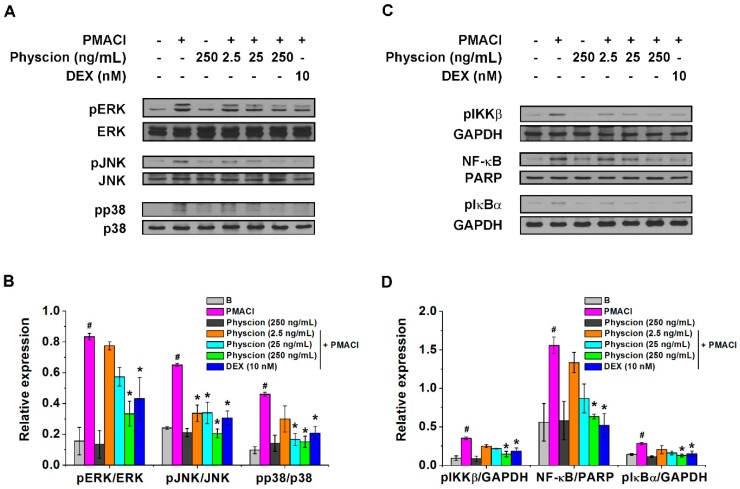
Physcion attenuates mitogen-activated protein kinase (MAPK)s and pIKKβ/NF-κB/pIkBα expressions in PMACI-stimulated HMC-1 cells. (**A**) HMC-1 cells (5 × 10^6^) were treated with Physcion or DEX for 1 h and then stimulated with PMACI for 30 min. Representative immunoblots are shown. (**B**) Each relative intensity of protein levels was quantified by densitometry and shown as pERK/ERK, pJNK/JNK, and pp38/p38. (**C**) HMC-1 cells (5 × 10^6^) were treated with Physcion or DEX for 1 h and then stimulated with PMACI for 1 h (pIKKβ) or 2 h (NF-κB and pIκBα). Representative immunoblots are shown. (**C, top**) Whole extract probed with anti-pIKKβ antibody. (**C, middle**) Nuclear extract probed with anti-NFκB p65 antibody. (**C, bottom**) Cytosolic extract probed with anti-pIκBα antibody. (**D**) Each relative intensity of protein levels was quantified by densitometry and shown as pIKKβ/GAPGH, NF-κB/PARP, and pIκBα/GAPGH. #*p* < 0.05 vs. a non-stimulated group. *p < 0.05 vs. a PMACI-stimulated group.

We next wished to identify whether physcion would modulate mitogen-activated protein kinase (MAPK)s and pIKKβ/NF-κB/pIkBα expression levels because TSLP levels secreted from HMC-1 cells are regulated via MAPKs/NF-κB signaling pathways [15,22]. The stimulation with PMACI induced significant increases in the expression levels of pERK, pJNK, and pp38 MAPKs compared to those of Blank group (non-stimulated group) (Figure 4A,B, *p* < 0.05). The treatment with physcion (250 ng/mL) significantly reduced the expression levels increased by the stimulation with PMACI (Figure 4A,B, *p* < 0.05). In addition, physcion (250 ng/mL) significantly suppressed the expression levels of pIKKβ, NF-κB, and pIkBα increased by the stimulation with PMACI (Figure 4C,D, *p* < 0.05). The regulatory effects of physcion (250 ng/mL) on expression levels of pERK/pJNK/pp38 and pIKKβ/NF-κB/pIkBα were similar to those of the DEX-treated group (Figure 4).

### 2.4. Physcion Attenuates TSLP Production in Anti-CD3 And Anti-CD28 Antibodies-Stimulated Splenocytes

The inflammatory cytokines in the spleen contribute to an AD-like allergic skin inflammation [23]. The stimulation with anti-CD3 and anti-CD28 antibodies activates purified splenic T cells and produces inflammatory cytokines [24,25]. Thus, we investigated whether physcion would regulate the production levels of TSLP, IL-4, IL-6, TNF-α, and IFN-γ secreted from anti-CD3 and anti-CD28 antibodies-stimulated splenocytes. Table 1 shows that the production levels of TSLP, IL-4, IL-6, TNF-α, and IFN-γ increased by anti-CD3 and anti-CD28 antibodies were significantly reduced by treatment with physcion (250 ng/mL) (*p* < 0.05). DEX also significantly inhibited the production levels of IL-4, IL-6, TNF-α, and IFN-γ secreted from the anti-CD3 and anti-CD28 antibodies-stimulated splenocytes (Table 1).

### 2.5. Physcion Relieves Pathological Changes of AD-Like Lesional Skin

**Figure 5:**
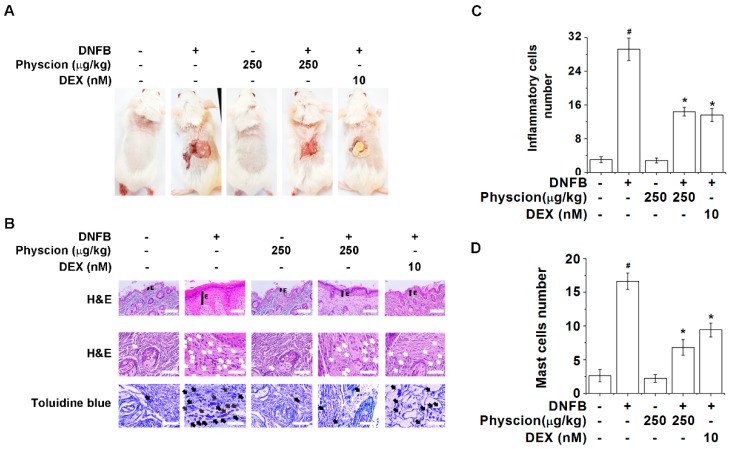
Physcion relieves pathological changes of AD-like lesional skin. (**A**) Representative dorsal skin photographs are shown. (**B, top**) Histological features of lesional skin tissues are shown. The sections from lesional skin were stained with H&E staining. E, epidermis. Magnification 200×, scale bar = 75 µm. (**B, middle**) Arrows indicate inflammatory cells. Magnification 400×, scale bar = 37 µm. (**B, bottom**) The sections from lesional skin were stained with toluidine blue staining. Arrows indicate mast cells. Magnification 400×, scale bar = 37 µm. The numbers of (**C**) inflammatory cells and (**D**) mast cells were quantified. # *p* < 0.05 vs. a vehicle group. * *p* < 0.05 vs. a DNFB control group. (n = 5).

We found that physcion inhibits TSLP levels as well as inflammatory cytokines levels in activated mast cells. Thus, we next wished to validate whether physcion could relieve AD-like symptoms using a DNFB-induced AD-like murine model. As shown in Figure 5A, repeated topical application of DNFB to the dorsal surface induced AD-like symptoms including erosion, erythema, scaling, and excoriation of lesional skin, however physcion markedly ameliorated these symptoms compared to those of the DNFB control group. Physcion also induced decreases in the thickness of the epidermis and infiltrations of inflammatory cells and mast cells in the lesional skin (Figure 5B–D, *p* < 0.05). DEX also ameliorated the pathological changes of the lesional skin similar to those observed in the physcion-treated group (Figure 5).

### 2.6. Physcion Inhibits Serum Histamine, IgE, And TSLP Levels in AD-Like Murine Model

**Figure 6:**
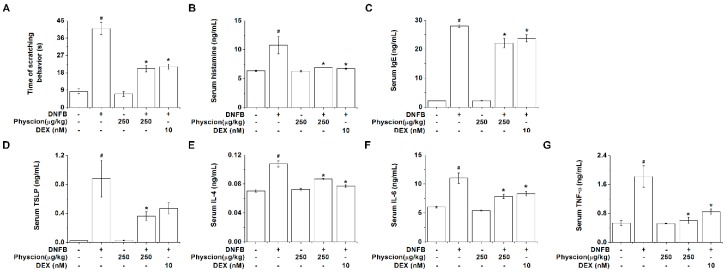
Physcion inhibits serum histamine, IgE, and TSLP levels in AD-like murine model. (**A**) The time of scratching behavior was measured for 10 min. (**B**) The serum histamine levels were analyzed with o-phthaldialdehyde spectrofluorometric procedure. The levels of (**C**) IgE, (**D**) TSLP, (**E**) IL-4, (**F**) IL-6, and (**G**) TNF-α in serum were detected by ELISA. # *p* < 0.05 vs. a vehicle group. * *p* < 0.05 vs. a DNFB control group.

Physcion significantly inhibited the time of scratching behavior in the AD-like murine model (Figure 6A, *p* < 0.05). Histamine, IgE, and TSLP induce scratching behaviors and accelerate the development of AD [26,27,28]. Thus, we measured the histamine, IgE, and TSLP levels in the serum of AD-like murine model. As shown in Figure 6B–D, physcion significantly suppressed the serum histamine, IgE, and TSLP levels (*p* < 0.05). Additionally, physcion significantly inhibited the serum IL-4, IL-6, and TNF-α levels (Figure 6E–G, *p* < 0.05). The regulatory effects of physcion on serum histamine, IgE, TSLP, IL-4, IL-6, and TNF-α levels were similar to those of DEX-treated group (Figure 6).

### 2.7. Physcion Reduces TSLP, IL-4, And IL-6 Expression Levels in Lesional Skin of DNFB-Induced AD-Like Murine Model

**Figure 7:**
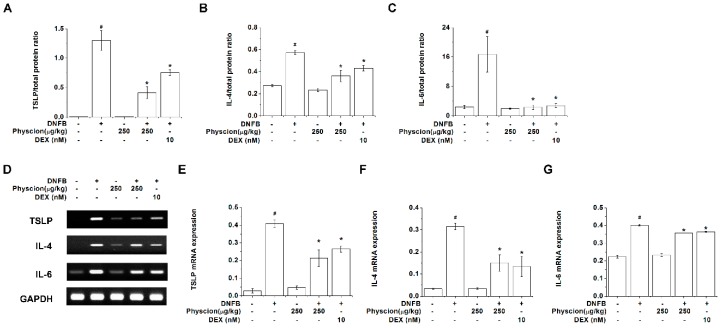
Physcion reduces TSLP, IL-4, and IL-6 expression levels in lesional skin. The protein expression levels of (**A**) TSLP, (**B**) IL-4, and (**C**) IL-6 in the lesional skin homogenate were detected by ELISA. (**D**) Representative reverse-transcription PCR gels are shown in the lesional skin tissues. The mRNA expression levels of (**E**) TSLP, (**F**) IL-4, and (**G**) IL-6 were normalized to the levels of GAPDH and expressed as fold induction relative to a vehicle group. Each mRNA level was quantified using the Image J software. # *p* < 0.05 vs. a vehicle group. * *p* < 0.05 vs. a DNFB control group.

Then, we further examined the regulatory effects of physcion on TSLP, IL-4, and IL-6 expression levels in the lesional skin tissues. The protein levels of TSLP, IL-4, and IL-6 were significantly reduced by the treatment with physcion (Figure 7A–C, *p* < 0.05). Physcion also significantly decreased the mRNA expression levels of TSLP, IL-4, and IL-6 in the lesional skin tissues (Figure 7D–G, *p* < 0.05). The regulatory effects of physcion on TSLP, IL-4, and IL-6 levels in the lesional skin tissues were similar to those of DEX-treated group (Figure 7).

### 2.8. Physcion Suppresses Caspase-1 Activation in AD-Like Lesional Skin

**Figure 8:**
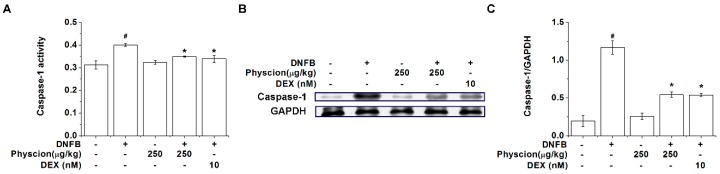
Physcion suppresses caspase-1 activation in AD-like lesional skin. (**A**) Caspase-1 activities in the lesional skin homogenate were analyzed using a caspase-1 colorimetric assay kit. (**B**) Representative immunoblots are shown in the lesional skin homogenate. (**C**) Each relative intensity of protein levels was quantified by densitometry and shown as caspase-1/GAPDH. # *p* < 0.05 vs. a vehicle group. * *p* < 0.05 vs. a DNFB control group.

We found that physcion inhibited TSLP levels via caspase-1 signaling pathways in activated HMC-1 cells. Thus, to further understand the roles of physcion on caspase-1 signaling during the development of AD, we finally examined a regulatory effect of physcion on the activity and protein expression level of caspase-1 in the lesional skin tissues. Figure 8A shows that the activity of caspase-1 was enhanced in the lesional skin tissues from AD-like mice, which was repressed by physcion (*p* < 0.05). Physcion also significantly reduced the protein expression level of caspase-1 in the lesional skin tissues (Figure 8B,C, *p* < 0.05). The regulatory effects of physcion on caspase-1 activation in the lesional skin tissues were similar to those of DEX-treated group (Figure 8).

## 3. Discussion

The crosslinking of FcεRI-bound IgE with multivalent antigen initiates activation of mast cells, then mast cell activation results in protein kinase C (PKC) activation as well as intracellular calcium elevation [29]. To produce similar conditions, we selected PMA to activate PKC and calcium ionophore to increase intracellular calcium level in this study. TSLP mRNA expression and production increased by stimulation with PMACI [15]. Increased TSLP expression was shown in skin lesions from patients with AD [30]. Furthermore, increased TSLP levels resulted in an exacerbation of scratching behavior in the AD model [31]. Deletion of TSLP improved AD-like skin lesions in mice [32]. Pretreatment with physcion decreased TSLP levels in HMC-1 cells, splenocytes, serum as well as skin lesions (Figure 2, Figure 6, Figure 7 and Table 1). Thus, we presume that physcion may be beneficial to prevent and/or treat atopic diseases. Patients with AD possess higher levels of proinflammatory cytokines such as IL-6, TNF-α, IL-1β, IL-4, and IFN-γ [33,34,35]. Our results showed that physcion suppresses the levels of IL-6, TNF-α, IL-1β, IL-4, and IFN-γ, suggesting the potential of physcion in the treatment of AD.

Han et al. [21] reported that calcium chelator decreases RIP2 levels in HMC-1 cells, indicating that RIP2 is a downstream factor of calcium. In addition, Humke et al. [36] reported that RIP2 activates caspase-1. An increment of intracellular calcium was inhibited by pretreatment with physcion in HMC-1 cells (Figure 2). Thus, we assume that physcion may decrease TSLP levels via inhibiting of calcium/RIP2/caspase-1 signal cascade in HMC-1 cells.

Caspase-1 is activated by proinflammatory stimuli [36]. Lots of research has shown that caspase-1 is activated by proinflammatory stimuli such as PMACI [37,38,39]. Caspase-1 overexpression leads to AD-like skin lesions in mice [40]. Downregulation of caspase-1 by inhibitor treatment ameliorates AD symptoms in mice [41]. Our results showed that physcion downregulates caspase-1 activation in HMC-1 cells as well as AD-like skin lesions (Figure 3 and Figure 8), presenting that physcion may ameliorate AD symptoms by the downregulation of caspase-1 activation.

The knock out of RIP2 shows decreased phosphorylation levels of ERK, JNK, and p38 MAPKs [42]. Treatment with caspase-1 inhibitor attenuates phosphorylation of p38, which suggests that caspase-1 is an upstream factor of p38 [43]. Furthermore, caspase-1 inhibition suppresses phosphorylation of ERK, JNK, and p38 MAPKs in human macrophages [44]. The results of the present study present that physcion suppresses the phosphorylation of ERK, JNK, and p38 in HMC-1 cells (Figure 4). Thus, we presume that suppression of TSLP by pretreatment with physcion, at least in part, may be mediated by RIP2/caspase-1/MAPKs signaling.

Lee and Ziegler [45] suggested that inducible expression of TSLP is mediated by NF-κB. Moon and Kim [15] also suggested that TSLP production is controlled by NF-κB in HMC-1 cells. Pretreatment with physcion suppressed phosphorylation of IKKβ and IκBα as well as activation of NF-κB (Figure 4), suggesting that physcion would suppress TSLP via inhibiting of NF-κB.

Histological AD features in human are epidermal thickening and inflammatory infiltrate [46]. Physcion ameliorates AD-like skin lesions, epidermal thickening, and inflammatory cell infiltration (Figure 5). Furthermore, mast cell infiltration is upregulated in the skin lesions of AD, which suggests that mast cells play a role in AD [47]. Mast cell infiltration into skin lesions was downregulated by the oral administration of physcion (Figure 5). Patients with AD showed higher histamine levels in serum compared with those in healthy subjects [48]. In addition, remarkable amelioration in the symptoms of AD resulted from anti-histamine treatment [48]. Our results showed that physcion reduces serum histamine levels and scratching behaviors (Figure 6). Therefore, we assume that physcion may be used as an anti-histamine therapy for AD. In this study, figures and tables use different concentrations of physcion, some use ng/mL, others μg/kg. While it is similar in biochemical experiments, it is not the same in case of animal experiments. Nevertheless, a lot of researchers use the same method in various models [49,50,51,52]. Thus, we used ng/mL or μg/kg to show the concentrations of physcion.

In conclusion, we showed that treatment with physcion decreases mRNA expression and production of TSLP, IL-6, TNF-α, and IL-1β in activated HMC-1 cells. Pretreatment with physcion downregulated the levels of intracellular calcium, activation of RIP2 and caspase-1, phosphorylation of p38, JNK, and ERK, activation of NF-κB, as well as phosphorylation of IKKβ and IκBα in activated HMC-1 cells. In addition, physcion reduced the production of TSLP, TNF-α, IL-4, IL-6, and IFN-γ from activated splenocytes. Oral administration of physcion improved AD-like skin lesions and reduced the levels of TSLP, IL-4, and IL-6, as well as caspase-1 activation in the skin lesions. In serum, histamine, IgE, TSLP, TNF-α, IL-6, and IL-4 levels were downregulated by physcion. These findings indicate that physcion could ameliorate AD-like skin lesions by inhibiting TSLP levels via caspase-1/MAPKs/NF-kB signalings, which would provide experimental evidence of the therapeutic potential of physcion for the treatment of AD.

## 4. Materials and Methods

### 4.1. Reagents

We purchased physcion (purity ≥ 98%, Figure 1A) from Cayman Chemical Company (Ann Arbor, MI, USA); Isocove’s Modified Dulbecco’s Medium (IMDM) from Gibco BRL (Grand Island, NY, USA); fetal bovine serum (FBS) from Welgene (Daegu, Korea); DEX, PMACI, 3-(4,5-dimethylthiazol-2-yl)-2,5-diphenyltetrazolium bromide (MTT), BAPTA-AM, Fura-2/AM, DNFB, and *O*-phthaldialdehyde from Sigma Chemical Co. (St. Louis, MO, USA); RPMI Media 1640 from Thermo Fisher Scientific Inc. (Waltham, MA, USA); anti-TSLP from R & D system Inc. (Minneapolis, MN, USA), IL-6 from Pharmingen (Sandiego, CA, USA), TNF-α (Pharmingen), IL-1β (R & D system Inc.), IL-4 (Pharmingen), IFN-γ (Pharmingen), and IgE antibodies (Pharmingen); anti- pERK, ERK, pJNK, JNK, pp38, p38, RIP2, caspase-1, pIKKβ, NF-κB, pIκBα, poly-ADP-ribose polymerase (PARP), GAPDH, and peroxidase-conjugated second antibodies from Santa Cruz Biotechnology (Santa Cruz, CA, USA); RIPA buffer from Cell signaling (Billerica, MA, USA).

### 4.2. Physcion Preparation

Physcion was dissolved in dimethyl sulfoxide (DMSO) according to a report by Shen et al. [53] and diluted with distilled water. The doses were decided according to a report by Shen et al. [53]. DEX was prepared according to a report by Chen et al. [54].

### 4.3. Cell Culture

HMC-1 cells were grown in IMDM supplemented with 10% heat-inactivated FBS and streptomycin (100 μg/mL)/penicillin (100 units/mL) at 37 °C in a 5% CO_2_ humidified incubator. HMC-1 cells were treated with 0.0025% DMSO (Blank group), physcion (2.5, 25, and 250 ng/mL), or DEX (10 nM) for 1 h prior to PMACI (PMA (20 nM) plus CI (1 µM)). The single cells (2.5 × 10^6^/mL) from spleen of normal BALB/c mice (Dae-Han Experimental Animal Center, Eumsung, Korea) were cultured in RPMI Media 1640 supplemented with 10% heat-inactivated FBS and streptomycin (100 μg/mL)/penicillin (100 units/mL) at 37 °C in a 5% CO_2_ humidified incubator. The splenocytes were treated with physcion or DEX and stimulated with immobilized anti-CD3 (2 mg/mL) plus soluble anti-CD28 (4 mg/mL) antibodies.

### 4.4. MTT Assay

An MTT assay was performed to evaluate cell viability. HMC-1 cells (3 × 10^4^ cells/well) were seeded into a 24-well plate and were then treated with different concentrations of physcion for 1 h. The cells were stimulated with PMACI for 7 h. 50 μL of MTT (5 mg/mL) solution was added to 500 μL of cell culture medium at 37 °C for 4 h. The crystallized formazan was then dissolved in DMSO. Optical density (O.D.) was detected at 540 nm by an enzyme-linked immunosorbent assay (ELISA) reader (Versa Max, Molecular Devices, Sunnyvale, CA, USA).

### 4.5. Intracellular Calcium Levels

HMC-1 cells (1 × 10^5^) were pretreated with Fura-2/AM (4 µM) in IMDM supplemented with 10% heat-inactivated FBS for 30 min. After the cells were washed with a calcium free medium containing EGTA (0.5 mM), the cells were treated with physcion or BAPTA-AM (10 µM) and stimulated with PMACI. The kinetics of intracellular calcium was measured for 100 s with a spectrofluorometer (excitation 360 nm, emission 450 nm, Thermo Fisher Scientific Inc., Shanghai, China).

### 4.6. Cytokines Assay

The levels of TSLP, IL-6, TNF-α, IL-1β, IL-4, IFN-γ, and IgE from HMC-1 cells supernatant, splenocytes supernatant, serum, or lesional skin homogenates were detected by ELISA according to the manufacturer’s instructions (R & D system Inc. and Pharmingen). The protein concentration was analyzed using a bicinchoninic acid protein assay kit.

### 4.7. Quantitative Real-Time Polymerase Chain Reaction (PCR) And Quantitative Reverse-Transcription PCR

Total RNA from HMC-1 cells or lesional skin tissues was isolated with an easy-BLUE™ RNA extraction kit (iNtRON Biotech, Sungnam, Korea). A cDNA synthesis kit (Bioneer Corporation, Daejeon, Korea) was used for synthesizing first-strand cDNA from total RNA. Real-time PCR for HMC-1 cells was performed using a SYBR Green master mix with an ABI StepOne real time-PCR System (Applied Biosystems, Foster City, CA, USA). Reverse-transcription PCR for lesional skin tissues was performed using an i-MAX™ II DNA polymerase kit (iNtRON Biotech, Sungnam, Korea) with a C1000 Touch Thermal Cycler (Bio-Rad Laboratories, Inc., Hercules, CA, USA). Primer sequences were as follows: hTSLP (5′ TAT GAG TGG GAC CAA AAG TAC CG 3′; 5′ GGG ATT GAA GGT TAG GCT CTG G 3′); hIL-6 (5′ AAA TTC GGT ACA TCC TCG ACG GCA 3′; 5′ AGT GCC TCT TTG CTG CTT TCA CAC 3′); hTNF-α (5′ AGG ACG AAC ATC CAA CCT TC 3′; 5′ TTT GAG CCA GAA GAG GTT GA 3′); hIL-1β (5′ AAA CAG ATG AAG TGC TCC TT 3′; 5′ TGG AGA ACA CCA CTT GTT GC 3′); hGAPDH (5′ TCG ACA GTC AGC CGC ATC TTC TTT 3′; 5′ ACC AAA TCC GTT GAC TCC GAC CTT 3′); mTSLP (5′ TGC AAG TAC TAG TAC GGA TGG GG C 3′; 5′ GGA CTT CTT GTG CCA TTT CCT GAG 3′); mIL-4 (5′ ACG GAG ATG GAT GTG CCA AA 3′; 5′ CGA GTA ATC CAT TTG CAT GA 3′); mIL-6 (5′ CGG GAT CCATGT TCC CTA CTT CAC AA 3′; 5′ CCC AAG CTT CTA CGT TTG CC 3′); mGAPDH (5′ GGC ATG GAC TGT GGT CAT GA 3′; 5′ TTC ACC ACC ATG GAG AAG GC 3′). Reverse-transcription PCR products were electrophoresed on a 1.5% ethidium bromide agarose gel. Each mRNA level was quantified using the Image J software (National Institute of Health, Bethesda, MD, USA).

### 4.8. Western Blotting

The HMC-1 cells lysed with RIPA buffer to prepare whole cell extracts and lesional skin homogenates were separated on 12% SDS-PAGE and transferred to nitrocellulose membranes. Equivalent amounts of protein were blocked with phosphate buffered saline with Tween 20 containing 5% non-fat dry milk and probed with primary antibodies against pERK, ERK, pJNK, JNK, pp38, p38, RIP2, caspase-1, pIKKβ, NF-κB, pIκBα, PARP, and GAPDH at 4 °C overnight. The membrane was then probed with peroxidase-conjugated second antibodies. Immunodetection was carried out using an enhanced chemiluminescence solution (DoGenBio Co., Seoul, Korea). Each band intensity was quantified using the Image J software (National Institute of Health).

### 4.9. Caspase-1 Activity Assay

The enzymatic activities of caspase-1 in HMC-1 cells lysates and lesional skin homogenates were analyzed with a caspase-1 assay kit according to the manufacturer’s instructions (R & D system Inc.).

### 4.10. Nuclear Extracts And Cytoplasmic Extracts

The pellet from HMC-1 cell was resuspended in 40 μL of cytoplasmic extract buffer (0.1 mM EDTA, 10 mM HEPES/KOH, 2 mM MgCl2, 1 mM dithiothreitol, 10 mM KCl, and 0.5 mM phenylmethylsulfonyl fluoride) for 15 min on ice and then lysed with 0.6 μL of 10% Nonidet P-40. After centrifuge, the cytoplasmic extract was removed from the pellet to a clean tube. The nuclei pellet was washed in 20 μL of cytoplasmic extract buffer and lysed with 40 μL of nuclear extract buffer (50 mM HEPES/KOH, 0.1 mM EDTA, 1 mM dithiothreitol, 50 mM KCl, 300 mM NaCl, 10% glycerol, and 0.5 mM phenylmethylsulfonyl fluoride) for 20 min on ice. After centrifuge, supernatant (nuclear fraction) was transfered to a clean tube.

### 4.11. Animals

All experiments were performed according to internationally-accepted standards for laboratory animal use and care, as found in the United States guidelines (NIH publication no. 85-23, revised in 1985). Studies involving animals was approved from the animal care committee of Kyung Hee University [KHUASP (SE)-18-022]. Female BALB/c mice of 8 weeks (Dae-Han Experimental Animal Center) were raised under conventional conditions (22 ± 1 °C, 40%–50% relative humidity, and 12:12 h light/dark cycle).

### 4.12. DNFB-Induced AD-Like Lesional Skin

DNFB-induced AD-like lesional skin was prepared as described previously [6] (Figure 1B). 100 μL of acetone or 0.15% DNFB was topically applied to shaved abdominal skin on 1st day. The shaved dorsal surface was applied with 50 μL of acetone as a vehicle group or 50 μL of DNFB as a control group on the eighth day and these applications were processed twice a week for 3 weeks. At the same time, 0.0025% DMSO (vehicle group), physcion (250 μg/kg), or DEX (10 nM) was orally administered to the mice three times a week for 3 weeks. For example, 200 μL of physcion solution (25 μg/mL) was orally administered to a mouse (20 g, body weight). Serum and lesional skin were collected 4 h after the last DNFB sensitization after anesthesia.

### 4.13. Histological Analysis

The lesional skin tissues fixed in 4% formalin were embedded in paraffin. Tissue slides (4 µm) were deparaffinized and rehydrated for staining with hematoxylin and eosin (H&E) or toluidine blue.

### 4.14. Histamine Assay

Serum histamine levels were analyzed according to o-phthaldialdehyde spectrofluorometric procedure, as previously described [55].

### 4.15. Statistics

The results are expressed as the mean ± standard error of mean (SEM). Statistical analysis was conducted using the IBM SPSS v23 statistics software (Armonk, NY, USA). An independent t-test was conducted for comparing differences between two groups (Blank group vs. PMACI control group or vehicle group vs. DNFB control group). ANOVA with Tukey post-hoc test was conducted for comparing differences among the PMACI control group/DNFB control group vs. the physcion or DEX-treated group. A *p* value <0.05 was considered significant.

## Figures and Tables

**Table 1:** Physcion attenuates thymic stromal lymphopoietin (TSLP) production in anti-CD3 and anti-CD28 antibodies-stimulated splenocytes.

	Blank	-	Physcion (250 ng/mL)	Physcion (2.5 ng/mL)	Physcion (25 ng/mL)	Physcion (250 ng/mL)	DEX (10 nM)
	-	CD3/CD28	-	CD3/CD28	CD3/CD28	CD3/CD28	CD3/CD28
TSLP (ng/mL)	0.16 ± 0.02	0.80 ± 0.14 ^#^	0.18 ± 0.01	0.65 ± 0.11	0.51 ± 0.08	0.25 ± 0.01 *	0.56 ± 0.11
IL-4 (ng/mL)	0.00 ± 0.00	0.60 ± 0.15 ^#^	0.00 ± 0.00	0.42 ± 0.02	0.31 ± 0.04	0.24 ± 0.01 *	0.03 ± 0.00 *
IL-6 (ng/mL)	0.02 ± 0.00	1.85 ± 0.05 ^#^	0.02 ± 0.00	1.55 ± 0.11 *	1.14 ± 0.02 *	1.03 ± 0.02 *	0.22 ± 0.01 *
TNF-α (ng/mL)	0.14 ± 0.02	0.56 ± 0.06 ^#^	0.11 ± 0.00	0.46 ± 0.05	0.30 ± 0.01 *	0.22 ± 0.01 *	0.29 ± 0.02 *
IFN-γ (ng/mL)	0.00 ± 0.00	35.95 ± 6.55 ^#^	0.00 ± 0.00	16.78 ± 1.00 *	10.99 ± 0.76 *	8.51 ± 0.69 *	4.85 ± 0.10 *

Splenocytes (2.5 × 10^6^/mL were stimulated with anti-CD3 plus anti-CD28 antibodies and treated with Physcion or DEX for 24 h. The production levels of inflammatory cytokines were detected by ELISA. Blank, non-stimulated cells; CD3/CD28, anti-CD3/anti-CD28 antibodies-stimulated cells. # *p* < 0.05 vs. a non-stimulated group. * *p* < 0.05 vs. an anti-CD3/CD28 antibodies-stimulated group.

## References

[1] Camargo Lopes de Oliveira L., Pierotti F.F., Mallozi M., Cocco R.R., Rosário N., Rugue Genov I., Åberg K.M., Borres M.P., Solé D. (2019). rBlo t 5 is a potential contributor to the severity of atopic dermatitis in a Brazilian population. Pediatr. Allergy Immunol..

[2] Ingordo V., Cazzaniga S., Naldi L., Perrucci S., Barbierato M., Crociata F., Cusano F. (2019). Atopic dermatitis in young adult italian males: Persistent and adult-onset varieties did not differ clinically and as for allergological variables. G. Ital. Dermatol. Venereol..

[3] Kircik L.H. (2019). Management of Atopic Dermatitis. J. Drugs Dermatol..

[4] Reed B., Blaiss M.S. (2018). The burden of atopic dermatitis. Allergy Asthma Proc..

[5] Barr T.P., Garzia C., Guha S., Fletcher E.K., Nguyen N., Wieschhaus A.J., Ferrer L., Covic L., Kuliopulos A. (2019). PAR2 Pepducin-Based Suppression of Inflammation and Itch in Atopic Dermatitis Models. J. Investig. Dermatol..

[6] Han N.R., Moon P.D., Kim H.M., Jeong H.J. (2018). Cordycepin ameliorates skin inflammation in a DNFB-challenged murine model of atopic dermatitis. Immunopharmacol. Immunotoxicol..

[7] Sasso O., Summa M., Armirotti A., Pontis S., De Mei C., Piomelli D. (2018). The *N*-Acylethanolamine Acid Amidase Inhibitor ARN077 Suppresses Inflammation and Pruritus in a Mouse Model of Allergic Dermatitis. J. Investig. Dermatol..

[8] Zhu Y., Pan W.H., Wang X.R., Liu Y., Chen M., Xu X.G., Liao W.Q., Hu J.H. (2015). Tryptase and protease-activated receptor-2 stimulate scratching behavior in a murine model of ovalbumin-induced atopic-like dermatitis. Int. Immunopharmacol..

[9] Schneider C., Döcke W.D., Zollner T.M., Röse L. (2009). Chronic mouse model of TMA-induced contact hypersensitivity. J. Investig. Dermatol..

[10] Han N.R., Oh H.A., Nam S.Y., Moon P.D., Kim D.W., Kim H.M., Jeong H.J. (2014). TSLP induces mast cell development and aggravates allergic reactions through the activation of MDM2 and STAT6. J. Investig. Dermatol..

[11] Han N.R., Moon P.D., Yoou M.S., Chang T.S., Kim H.M., Jeong H.J. (2018). Effect of massage therapy by VOSKIN 125+ painkiller® on inflammatory skin lesions. Dermatol. Ther..

[12] Schneider K.S., Groß C.J., Dreier R.F., Saller B.S., Mishra R., Gorka O., Heilig R., Meunier E., Dick M.S., Ćiković T. (2017). The Inflammasome Drives GSDMD-Independent Secondary Pyroptosis and IL-1 Release in the Absence of Caspase-1 Protease Activity. Cell Rep..

[13] Han N.R., Moon P.D., Kim N.R., Kim H.Y., Jeong H.J., Kim H.M. (2017). Schisandra chinensis and Its Main Constituent Schizandrin Attenuate Allergic Reactions by Down-Regulating Caspase-1 in Ovalbumin-Sensitized Mice. Am. J. Chin. Med..

[14] Błażejewski A.J., Thiemann S., Schenk A., Pils M.C., Gálvez E.J.C., Roy U., Heise U., de Zoete M.R., Flavell R.A., Strowig T. (2017). Microbiota Normalization Reveals that Canonical Caspase-1 Activation Exacerbates Chemically Induced Intestinal Inflammation. Cell Rep..

[15] Moon P.D., Kim H.M. (2011). Thymic stromal lymphopoietin is expressed and produced by caspase-1/NF-κB pathway in mast cells. Cytokine.

[16] Mueller S.O., Schmitt M., Dekant W., Stopper H., Schlatter J., Schreier P., Lutz W.K. (1999). Occurrence of emodin, chrysophanol and physcion in vegetables, herbs and liquors. Genotoxicity and anti-genotoxicity of the anthraquinones and of the whole plants. Food Chem. Toxicol..

[17] Qin X., Peng Y., Zheng J. (2018). In Vitro and in Vivo Studies of the Electrophilicity of Physcion and its Oxidative Metabolites. Chem. Res. Toxicol..

[18] Pan X.P., Wang C., Li Y., Huang L.H. (2018). Physcion induces apoptosis through triggering endoplasmic reticulum stress in hepatocellular carcinoma. Biomed. Pharmacother..

[19] Pan X., Wang H., Tong D., Wang C., Sun L., Zhao C., Li Y., Zhu L., Wu D. (2016). Physcion induces apoptosis in hepatocellular carcinoma by modulating miR-370. Am. J. Cancer Res..

[20] Chen X., Gao H., Han Y., Ye J., Xie J., Wang C. (2015). Physcion induces mitochondria-driven apoptosis in colorectal cancer cells via downregulating EMMPRIN. Eur. J. Pharmacol..

[21] Han N.R., Kim H.M., Jeong H.J. (2012). Thymic stromal lymphopoietin is regulated by the intracellular calcium. Cytokine.

[22] Kim M.H., Seo J.H., Kim H.M., Jeong H.J. (2016). Aluminum-doped zinc oxide nanoparticles attenuate the TSLP levels via suppressing caspase-1 in activated mast cells. J. Biomater. Appl..

[23] Debnath T., Lee Y.M., Lim J.H., Lim B.O. (2018). Anti-allergic and anti-atopic dermatitis effects of Gardenia Fructus extract. Food Agric. Immunol..

[24] Gonzalez J., Orlofsky A., Prystowsky M.B. (2003). A1 is a growth-permissive antiapoptotic factor mediating postactivation survival in T cells. Blood.

[25] Han N.R., Moon P.D., Kim H.M., Jeong H.J. (2012). Effect of Pyeongwee-San (KMP6) on 2,4-dinitrofluorobenzene-induced atopic dermatitis-like skin lesions in NC/Nga mice. Life Sci..

[26] Weber M.B., Petry V., Weis L., Mazzotti N.G., Cestari T.F. (2005). Evaluating the relation between pruritus, serum IgE levels and severity of clinical manifestations in atopic dermatitis patients. An. Bras. Dermatol..

[27] Wilson S.R., Thé L., Batia L.M., Beattie K., Katibah G.E., McClain S.P., Pellegrino M., Estandian D.M., Bautista D.M. (2013). The epithelial cell-derived atopic dermatitis cytokine TSLP activates neurons to induce itch. Cell.

[28] Wong L.S., Wu T., Lee C.H. (2017). Inflammatory and Noninflammatory Itch: Implications in Pathophysiology-Directed Treatments. Int. J. Mol. Sci..

[29] Moon P.D., Han N.R., Lee J.S., Kim H.M., Jeong H.J. (2018). Effects of Linalyl Acetate on Thymic Stromal Lymphopoietin Production in Mast Cells. Molecules.

[30] Ziegler S.F. (2010). The role of thymic stromal lymphopoietin (TSLP) in allergic disorders. Curr. Opin. Immunol..

[31] Jang H., Matsuda A., Jung K., Karasawa K., Matsuda K., Oida K., Ishizaka S., Ahn G., Amagai Y., Moon C. (2016). Skin pH is the Master Switch of Kallikrein 5-Mediated Skin Barrier Destruction in a Murine Atopic Dermatitis Model. J. Investig. Dermatol..

[32] Moon P.D., Han N.R., Kim H.M., Jeong H.J. (2018). High-Fat Diet Exacerbates Dermatitis through Up-Regulation of TSLP. J. Investig. Dermatol..

[33] Szymanski L., Cios A., Lewicki S., Szymanski P., Stankiewicz W. (2018). Fas/FasL pathway and cytokines in keratinocytes in atopic dermatitis—Manipulation by the electromagnetic field. PLoS ONE.

[34] Szegedi K., Lutter R., Res P.C., Bos J.D., Luiten R.M., Kezic S., Middelkamp-Hup M.A. (2015). Cytokine profiles in interstitial fluid from chronic atopic dermatitis skin. J. Eur. Acad. Dermatol. Venereol..

[35] Batista D.I., Perez L., Orfali R.L., Zaniboni M.C., Samorano L.P., Pereira N.V., Sotto M.N., Ishizaki A.S., Oliveira L.M., Sato M.N. (2015). Profile of skin barrier proteins (filaggrin, claudins 1 and 4) and Th1/Th2/Th17 cytokines in adults with atopic dermatitis. J. Eur. Acad. Dermatol. Venereol..

[36] Humke E.W., Shriver S.K., Starovasnik M.A., Fairbrother W.J., Dixit V.M. (2000). ICEBERG: A novel inhibitor of interleukin-1beta generation. Cell.

[37] Moon P.D., Han N.R., Ryu K.J., Kang S.W., Go J.H., Jang J.B., Choi Y., Kim H.M., Jeong H.J. (2016). A novel compound 2-(4-{2-[(phenylthio)acetyl]carbonohydrazonoyl}phenoxy)acetamide downregulates TSLP through blocking of caspase-1/NF-κB pathways. Int. Immunopharmacol..

[38] Han N.R., Moon P.D., Kim H.M., Jeong H.J. (2014). Tryptanthrin ameliorates atopic dermatitis through down-regulation of TSLP. Arch. Biochem. Biophys..

[39] Moon P.D., Choi I.H., Kim H.M. (2012). Epigallocatechin-3-*O*-gallate inhibits the production of thymic stromal lymphopoietin by the blockade of caspase-1/NF-κB pathway in mast cells. Amino Acids.

[40] Kakeda M., Yamanaka K., Kitagawa H., Tsuda K., Akeda T., Kurokawa I., Gabazza E.C., Mizutani H. (2012). Heat-killed bacillus Calmette-Guérin and Mycobacterium kansasii antigen 85B combined vaccination ameliorates dermatitis in a mouse model of atopic dermatitis by inducing regulatory T cells. Br. J. Dermatol..

[41] Hiramoto K., Yamate Y., Yokoyama S. (2018). Ultraviolet B eye irradiation aggravates atopic dermatitis via adrenocorticotropic hormone and NLRP3 inflammasome in NC/Nga mice. Photodermatol. Photoimmunol. Photomed..

[42] Kobayashi K., Inohara N., Hernandez L.D., Galán J.E., Núñez G., Janeway C.A., Medzhitov R., Flavell R.A. (2002). RICK/Rip2/CARDIAK mediates signalling for receptors of the innate and adaptive immune systems. Nature.

[43] Zhang F., Wang L., Wang J.J., Luo P.F., Wang X.T., Xia Z.F. (2016). The caspase-1 inhibitor AC-YVAD-CMK attenuates acute gastric injury in mice: Involvement of silencing NLRP3 inflammasome activities. Sci. Rep..

[44] Hedl M., Abraham C. (2011). Distinct roles for Nod2 protein and autocrine interleukin-1beta in muramyl dipeptide-induced mitogen-activated protein kinase activation and cytokine secretion in human macrophages. J. Biol. Chem..

[45] Lee H.C., Ziegler S.F. (2007). Inducible expression of the proallergic cytokine thymic stromal lymphopoietin in airway epithelial cells is controlled by NFkappaB. Proc. Natl. Acad. Sci. USA.

[46] Rizzo J.M., Oyelakin A., Min S., Smalley K., Bard J., Luo W., Nyquist J., Guttman-Yassky E., Yoshida T., De Benedetto A. (2016). ΔNp63 regulates IL-33 and IL-31 signaling in atopic dermatitis. Cell Death Differ..

[47] Choi E.J., Iwasa M., Han K.I., Kim W.J., Tang Y., Hwang Y.J., Chae J.R., Han W.C., Shin Y.S., Kim E.K. (2016). Heat-Killed Enterococcus faecalis EF-2001 Ameliorates Atopic Dermatitis in a Murine Model. Nutrients.

[48] Imaizumi A., Kawakami T., Murakami F., Soma Y., Mizoguchi M. (2003). Effective treatment of pruritus in atopic dermatitis using H1 antihistamines (second-generation antihistamines): Changes in blood histamine and tryptase levels. J. Dermatol. Sci..

[49] Tian H., Liu Z., Pu Y., Bao Y. (2019). Immunomodulatory effects exerted by Poria Cocos polysaccharides via TLR4/TRAF6/NF-κB signaling in vitro and in vivo. Biomed. Pharmacother..

[50] Liu G., Park Y.J., Tsuruta Y., Lorne E., Abraham E. (2009). p53 Attenuates lipopolysaccharide-induced NF-kappaB activation and acute lung injury. J. Immunol..

[51] Lee S.A., Moon S.M., Han S.H., Hwang E.J., Hong J.H., Park B.R., Choi M.S., Ahn H., Kim J.S., Kim H.J. (2018). In Vivo and In Vitro Anti-Inflammatory Effects of Aqueous Extract of Anthriscus sylvestris Leaves. J. Med. Food..

[52] Hwang D.S., Gu P.S., Kim N., Jang Y.P., Oh M.S. (2018). Effects of Rhei Undulati Rhizoma on lipopolysaccharide-induced neuroinflammation in vitro and in vivo. Environ. Toxicol..

[53] Shen M.Y., Liu Y.J., Don M.J., Liu H.Y., Chen Z.W., Mettling C., Corbeau P., Chiang C.K., Jang Y.S., Li T.H. (2011). Combined phytochemistry and chemotaxis assays for identification and mechanistic analysis of anti-inflammatory phytochemicals in Fallopia japonica. PLoS ONE.

[54] Chen X., Murakami T., Oppenheim J.J., Howard O.M.Z. (2004). Differential response of murine CD4+CD25+ and CD4+CD25− T cells to dexamethasone-induced cell death. Eur. J. Immunol..

[55] Moon P.D., Choi I.S., Go J.H., Lee B.J., Kang S.W., Yoon S., Han S.J., Nam S.Y., Oh H.A., Han N.R. (2013). Inhibitory effects of BiRyuChe-bang on mast cell-mediated allergic reactions and inflammatory cytokines production. Am. J. Chin. Med..

